# miR-210-5p promotes epithelial–mesenchymal transition by inhibiting PIK3R5 thereby activating oncogenic autophagy in osteosarcoma cells

**DOI:** 10.1038/s41419-020-2270-1

**Published:** 2020-02-05

**Authors:** Wei Liu, Dongdong Jiang, Fangyi Gong, Yumin Huang, Yongjun Luo, Yuluo Rong, Jiaxing Wang, Xuhui Ge, Chengyue Ji, Jin Fan, Weihua Cai

**Affiliations:** Department of Orthopaedics, the First Affiliated Hospital of Nanjing Medical University, Nanjing, Jiangsu 210029 China

**Keywords:** Bone cancer, Autophagy

## Abstract

Osteosarcoma (OS) is a malignant bone tumor which occurs mainly in adolescents with frequent pulmonary metastasis and a high mortality rate. Accumulating evidence has indicated that microRNAs (miRNAs) play a vital role in various tumors by modulating target genes as well as signal pathways, and aberrant expression of miRNAs may contribute to OS progression. This study aimed to determine the association between miR-210-5p expression and OS progression and to investigate its potential underlying mechanism. Using reverse transcription-polymerase chain reaction (RT-PCR), miR-210-5p was found to be upregulated in clinical OS specimens and cell lines. Further functional analysis demonstrated that miR-210-5p promoted epithelial–mesenchymal transition (EMT) and induced oncogenic autophagy. Luciferase reporter assay, RNA-ChIP, and western blot analysis confirmed that PIK3R5, an essential regulator in the AKT/mTOR signaling pathway, is a target downstream gene of miR-210-5p. Overexpression or knockdown of PIK3R5 reversed the functional role of overexpression or knockdown of miR-210-5p, respectively. Silencing autophagy-related gene 5 (ATG5) abolished the functional effects of miR-210-5p upregulation or PIK3R5 knockdown in OS cells. In vivo, miR-210-5p overexpression promoted OS tumor growth and pulmonary metastasis. Taken together, our results demonstrated that miR-210-5p promoted EMT and oncogenic autophagy by suppressing the expression of PIK3R5 and regulating the AKT/mTOR signaling pathway. Therefore, inhibition of miR-210-5p may represent a promising treatment for OS.

## Introduction

Osteosarcoma (OS), a primary bone tumor arising from mesenchymal cells, has an extremely high fatality rate worldwide^1^. Regardless of the number of treatments including surgery supplemented by chemotherapy, the 5-year survival rate is <70% due to tumor metastasis as well as drug resistance^2^. Indeed, the overall survival rate in OS individuals with metastasis or recurrent disease is extremely low at ~10–20%, due to limited effective treatments^3,4^. Therefore, it is essential to identify the underlying mechanisms of OS metastasis and recurrence in order to develop effective and comprehensive treatments.

During the development of malignant tumors with metastasis, the EMT process plays a crucial role. EMT is a cellular process, which is characterized by cells losing epithelial characteristics and acquiring mesenchymal properties, which may contribute to enhanced OS tumorigenesis and metastasis^5–7^. Therefore, suppressing the progression of EMT may be a potentially effective way of treating OS.

MicroRNAs (miRNAs), a family of endogenous small noncoding RNAs, have been proven to regulate target mRNA expression by binding to the 3′-untranslated regions (3′-UTR) directly, and eventually promote target mRNA degradation or translational inhibition^8–10^. Accumulating evidence suggests that miRNAs act as oncogenes in various tumors^11,12^. Recent studies also identified miRNAs which may regulate cell proliferation, metastasis, and apoptosis in the development of OS^13–16^. MiR-210-5p, an onco-miR, which was reported to be increased in several malignant tumors, was shown to be significantly correlated with poor clinical outcomes^17–20^. However, the detailed functions of miR-210-5p in OS have not been investigated, and are thus not fully understood.

Autophagy, which is recognized as an intracellular degradation program which delivers selective cytoplasmic proteins and abnormal organelles to lysosomes for degradation, has important effects in various malignant tumors^21–23^. The exact role of autophagy in the progression of malignant tumors is controversial^24–26^. On the one hand, it has been proved that autophagy can enhance the viability of cancer cells, promote tumor metastasis by EMT, and promote drug resistance^27,28^. On the other hand, many studies have reported that autophagy can induce autophagic cell death of tumor cells and inhibit tumorigenesis and metastasis^29–31^. In recent years, it has been shown that autophagy can be regulated by miRNAs in several malignant tumors, and these miRNAs can act as promotors or inhibitors in the development of malignant tumors^32–34^. However, whether miR-210-5p can regulate autophagy and the underlying interaction between autophagy and EMT in OS metastasis remains to be examined in depth.

In this study, we demonstrated that miR-210-5p was significantly upregulated in OS and promoted EMT. It was found that the upregulation of miR-210-5p promoted EMT and oncogenic autophagy in OS by downregulating the expression of PIK3R5 and regulating the AKT/mTOR signaling pathway.

## Methods

### Clinical tissue specimens

This study was approved by the institutional review board and the ethics committee of the First Affiliated Hospital of Nanjing Medical University, and written informed consent was obtained from all patients. Sixty-two pairs of OS tissues and matched adjacent normal tissues were obtained during surgery from the Department of Orthopedics, the First Affiliated Hospital of Nanjing Medical University based on accepted radiological and pathological criteria. The tissue specimens were snap-frozen in liquid nitrogen and stored at −80 °C until RNA and protein extraction. Patient clinicopathological and demographic information are shown in Supplementary Table S1.

### Cell culture

The human OS cell lines including HOS, Saos-2, SW1353, U2OS, and MG63, and the normal human osteoblast cell line hFOB1.19 were obtained from the American Type Culture Collection (ATCC, Manassas, VA, USA). For each experiment, OS cells were cultured in Dulbecco’s Modified Eagle’s Medium (DMEM) supplemented with 10% fetal bovine serum (Gibco Laboratory, Grand Island, NY, USA), 1% penicillin and streptomycin.

### Vector constructs, lentivirus production, and cell transfection

LV2-has-miR-210-5p-mimic vector (LV-miR-210-5p) and the LV2- has-miR-210-5p-inhibitor vector (ANTI-miR-210-5p) were constructed using lentiviral vectors (GenePharma, Shanghai, China). Negative controls with the LV2 empty lentivirus (LV-NC and ANTI-NC) were also constructed. We infected OS cells grown to 40–50% confluence using lentiviral vectors at an appropriate multiplicity of infection. Puromycin (5 μg/ml, Sigma Aldrich) was used in selected transfected OS cells to generate stable OS cell lines. Vectors for overexpressing and downregulating target human PIK3R5 using lentiviral gene transfer and containing the puromycin-resistance sequence were constructed by GenePharma (Shanghai, China), and the scrambled lentiviral construct was used as a negative control. OS cells were transfected with the lentiviral vectors (Vector, PIK3R5, shNC, and shPIK3R5). Quantitative reverse transcription-polymerase chain reaction (qRT-PCR) and western blot analysis were then used to confirm PIK3R5 expression.

### RNA extraction and qRT-PCR

TRIzol reagent (Invitrogen, Carlsbad, CA, USA) was used to extract the total RNA from cells and specimens according to the manufacturer’s protocol. The quality and concentration of RNA were detected using a NanoDrop spectrophotometer (ND-100, Thermo Fisher Scientific). MiRNA and mRNA reverse transcription were conducted with the Hairpin-it^TM^ miRNA qPCR Quantitation Kit (GenePharma, China) and PrimeScript RT Master Mix Kit (RR036A, TaKaRa), respectively. RT-PCR was performed with the SYBR Green PCR master mix (Applied Biosystems, Foster City, CA, USA) on an ABI 7900 fast real-time PCR system (Applied Biosystems, Carlsbad, CA, USA). Expression levels of miR-210-5p and PIK3R5 were normalized to the internal controls (U6 and GAPDH), and the relative expression levels were evaluated using the 2^−ΔΔCT^ method. The specific primers used in our study were purchased from RiboBio Co, Ltd. (Guangzhou, China). The primer sequences are listed in Supplementary Table S2.

### Fluorescence in situ hybridization (FISH)

The expression of miR-210-5p in OS specimens and adjacent normal tissues was detected by fluorescence in situ hybridization (FISH). Probes against miR-210-5p were synthesized by BioSense (Guangzhou, China). FISH was performed according to the manufacturer’s protocol. Briefly, frozen human OS specimens were fixed with 4% paraformaldehyde for 20 min, digested with proteinase K for 3 min, and then continually dehydrated in 70, 85, and 100% ethanol for 5 min. Probes were denatured at 78 °C for 5 min, and then hybridized with sections in a humidification chamber at 42 °C overnight. Finally, the sections were washed three times with PBS and then stained with DAPI (Thermo Fisher Scientific) for 15 min, and fluorescent images were acquired under a fluorescence microscope (AxioVertA1 and ImagerA2).

### Invasion assay

Cell-invasion capacity was measured using 24-well BD Matrigel invasion chambers (BD Biosciences) according to the manufacturer’s instructions. Briefly, for the invasion assays, ~2 × 10^4^ OS cells were seeded on the upper well of the invasion chamber in DMEM medium without FBS, while the lower chamber well contained DMEM supplemented with 10% FBS to activate invasion. After 24 h, non-invading upper cells were wiped with a cotton swab. Cells which invaded the lower chamber were fixed with 4% paraformaldehyde and stained with 0.5% crystal violet for 30 min. The invasive cells were counted in three random microscopic views and photographed.

### Wound-healing assay

Cells were cultured in six-well plates and grown to 80–90% confluence. The cells were then scratched in the central area of the confluent culture using a fine pipette tip, and wound healing was observed at 0 and 24 h after injury.

### 3D spheroid BME cell-invasion assay

For 3D spheroid BME cell-invasion assays, established OS cells were seeded at a density of 2 × 10^4^ cells/ml in a 96-well ultralow adherence plate (#7007, Costar). The cells were induced to aggregate into a multicellular spheroid with an estimated density of 2000 cells after 96 h followed by the addition of Matrigel to the wells. The motion of these multicellular spheroid cells was then observed using fluorescence microscopy after 48 h.

### Cell counting kit-8 assay (CCK-8) and colony-formation assay

Transfected OS cells were cultured in 96-well plates (2000 cells with 100 μL culture medium per well) and incubated for 24, 48, 72, 96, and 120 h. Cell proliferation was analyzed by the CCK-8 assay (Dojindo, Japan) according to the manufacturer’s instructions. Optical absorbance was measured at 450 nm using a microplate reader (ELx800; Bio-Tek).

For the colony-formation assay, cells were cultured in six-well plates at a density of 500 cells/well and cultured in the medium with 10% FBS for 2 weeks. Subsequently, the cells were fixed and stained with crystal violet. The colonies were monitored and counted under a light microscope.

### Flow cytometric analysis

Transfected OS cells were collected and washed with pre-cold PBS twice. Then cell apoptosis was evaluated by double staining with FITC-conjugated Annexin V and propidium iodide (PI) (MultiSciences, Hangzhou, China) for 20 min in the dark. After washing three times with PBS, cells were immediately analyzed using a flow cytometer (FACSCalibur, BD Biosciences).

### Autophagosome detection by GFP-mRFP-LC3

A GFP-mRFP-LC3 (Obio, Shanghai, China) lentivirus was used for autophagosome detection. OS cells were transfected with a GFP-mRFP-LC3 lentivirus according to the manufacturer’s instructions. HOS and MG63 cells were fixed in 4% paraformaldehyde, and the nucleus stained with DAPI. The location and quantitation of autophagosomes were then observed using confocal microscopy (Zeiss, Oberkochen, Germany, LSM 510). Autophagosomes were labeled red and green (yellow fluorescence), whereas autophagic lysosomes were labeled red.

### Transmission electron microscopy (TEM)

The cells were fixed in precooled 2% glutaraldehyde in 0.1 M cacodylate buffer at 4 °C overnight and then exposed to phosphate buffer containing 1% osmium tetroxide for 1 h. After being dehydrated in different concentrations of acetone, the cells were infiltrated and embedded into Epon. The embedded materials were sectioned and stained with 3% uranyl acetate and lead citrate. Images were then obtained under an electron microscope (FEI Tecnai, Hillsboro, OR, USA).

### Luciferase reporter assay

Sequences corresponding to the 3′-UTR of PIK3R5 mRNA and containing the wild-type (WT) or mutated (MUT) miR-210-5p-binding sequence were synthesized by GeneScript (Nanjing, China). These sequences were cloned into the FseI and XbaI restriction sites of the pGL3 luciferase control reporter vector (Promega, Madison, WI, USA) to generate the PIK3R5 3′-UTR reporter constructs (pGL3-WT-PIK3R5 and pGL3-MUT-PIK3R5). HOS and MG63 cells transfected with LV-miR-210-5p or LV-NC were seeded into 96-well plates and co-transfected with 100 ng of pGL3-WT-PIK3R5 or pGL3-MUT-PIK3R5 3′-UTR. Firefly and Renilla luciferase signals were determined using a Dual-Luciferase® Assay Kit (Promega, Madison, WI, USA).

### Isolation of RISC-associated RNA

HOS and MG63 cells overexpressing miR-210-5p or negative control were fixed with 1% formaldehyde, followed by chromatin fragmentation, lysed in NETN buffer, and then incubated with Dynabeads Protein A (Invitrogen) supplemented with clone 2A8 antibody (Millipore), anti-Pan-Ago, or IgG control for immunoprecipitation. The immunoprecipitated RNA was released by proteinase K digestion, and extracted by phenol/chloroform/isopropyl alcohol. RNA was isolated by glycogen ethanol precipitation and treated with DNase I.

### Western blot

Western blot analysis was performed as described previously^35–37^. Anti-PIK3R5, N-cadherin, E-cadherin, Vimentin, MMP2, Beclin1, p62, ATG5, LC3-II, Bax, Bcl2, cleaved caspase-3, and GAPDH were purchased from Abcam (Cambridge, UK), and anti-p-AKT, AKT, p-mTOR, and mTOR were obtained from Cell Signaling Technology.

### Immunohistochemistry

All specimens were fixed in 4% paraformaldehyde and embedded in paraffin. The paraffin was then cut into 4-μm sections and incubated overnight with the primary antibody for PIK3R5, p62, vimentin, and ATG5 (Abcam, Cambridge, UK). The sections were then incubated with the secondary antibody for 1 h and stained using 3,3-diaminobenzidine (DAB) solution for 3 min. Three fields were selected to measure the percentage of positive tumors and staining intensities.

### Animal experiments

The animal studies were approved by the Institutional Animal Care and Use Committee of the First Affiliated Hospital of Nanjing Medical University. Four-week-old nude mice (BALB/c nude mice) used for tumor growth assays in vivo were purchased from the Animal Model Institute of Nanjing University (Nanjing, China). The nude mice were randomly divided into four groups (*n* = 6 per group). Stably transfected cells labeled with firefly luciferase (2 × 10^6^ cells) in 100 μL PBS were subcutaneously injected into the nude mice. The same nude mice and cell types as above were used for tumor metastasis assays. PBS containing 2 × 10^6^ cells was injected into nude mice via the caudal vein. The progression of xenograft growth and metastases were imaged using the IVIS200 imaging system (Caliper Life Sciences, Waltham, MA, USA).

### Statistical analysis

All experiments were performed in triplicate, and data are expressed as the mean ± standard deviation. The χ^2^ test was used to analyze the association between miR-210-5p and PIK3R5 expression level and clinicopathological features. One-way analysis of variance (ANOVA) was used for multiple group comparisons, and the Student’s *t* test was used to compare two groups. Statistical analyses were performed using SPSS v. 22.0 (SPSS Inc., Chicago, IL, USA). *P* < 0.05 was considered statistically significant.

## Results

### Upregulation of miR-210-5p in clinical OS specimens and cell lines

**Fig. 1:**
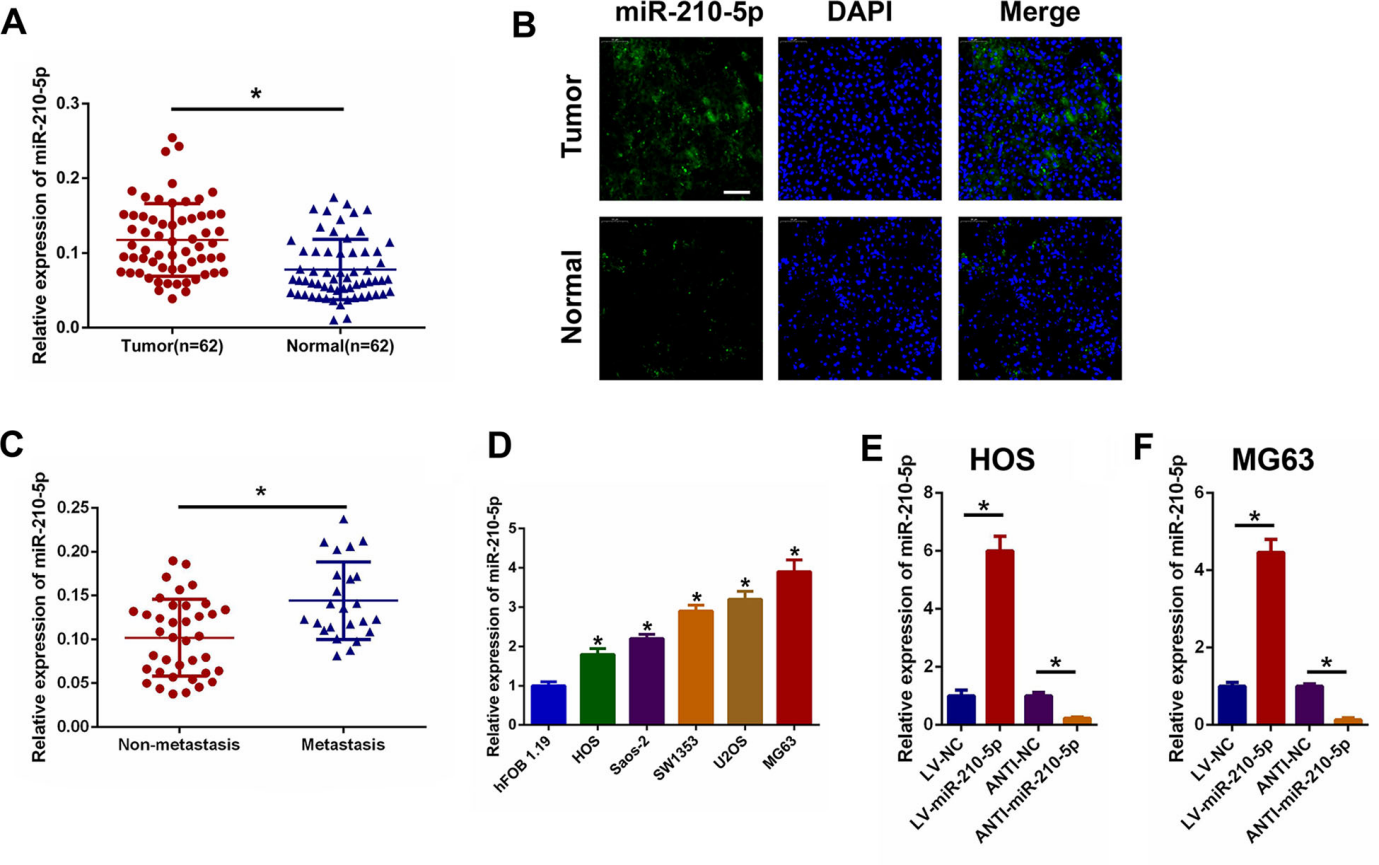
miR-210-5p is upregulated in clinical OS specimens and cell lines. **a** The expression of miR-210-5p in 62 pairs of clinical OS specimens and matched adjacent normal tissues. **b** Representative FISH images of miR-210-5p in clinical OS specimens and matched adjacent normal tissues. Scale bar = 50 μm. **c** The expression of miR-210-5p in the metastasis group compared with the non-metastasis group. **d** The relative expression of miR-210-5p in OS cells and the hFOB 1.19 cell line. **e**, **f** The expression of miR-210-5p in HOS and MG63 cells transfected with LV-miR-210-5p or ANTI-miR-210-5p.

First, we assessed the expression of miR-210-5p in 62 paired OS specimens and matched adjacent normal specimens. It was found the expression level of miR-210-5p was significantly upregulated in OS tissues compared with adjacent normal tissues (Fig. 1a). FISH was then used to detect the miR-210-5p expression level, and the results shown in Fig. 1b confirmed the above RT-PCR results. It was also found that miR-210-5p expression was higher in the metastasis group compared with the non-metastasis group (Fig. 1c). The representative radiological images of OS patients with or without pulmonary metastasis are shown in Supplementary Fig. S1. In OS cell lines, including HOS, Saos-2, SW1353, U2OS, and MG63, the expression level of miR-210-5p was upregulated in OS cell lines when compared with the normal human osteoblast cell line hFOB 1.19 (Fig. 1d). In addition, the expression level of miR-210-5p was obtained from the GEO online database and confirmed that the expression of miR-210-5p was higher in OS cell lines (Supplementary Fig. S2A). Furthermore, we analyzed the relationship between the expression level of miR-210-5p and the clinicopathological characteristics in OS patients (Supplementary Table S1). The expression level of miR-210-5p was found to be significantly positively correlated with TNM stage, lung metastasis, and tumor size.

### miR-210-5p promotes tumor invasion and migration in OS cells

**Fig. 2:**
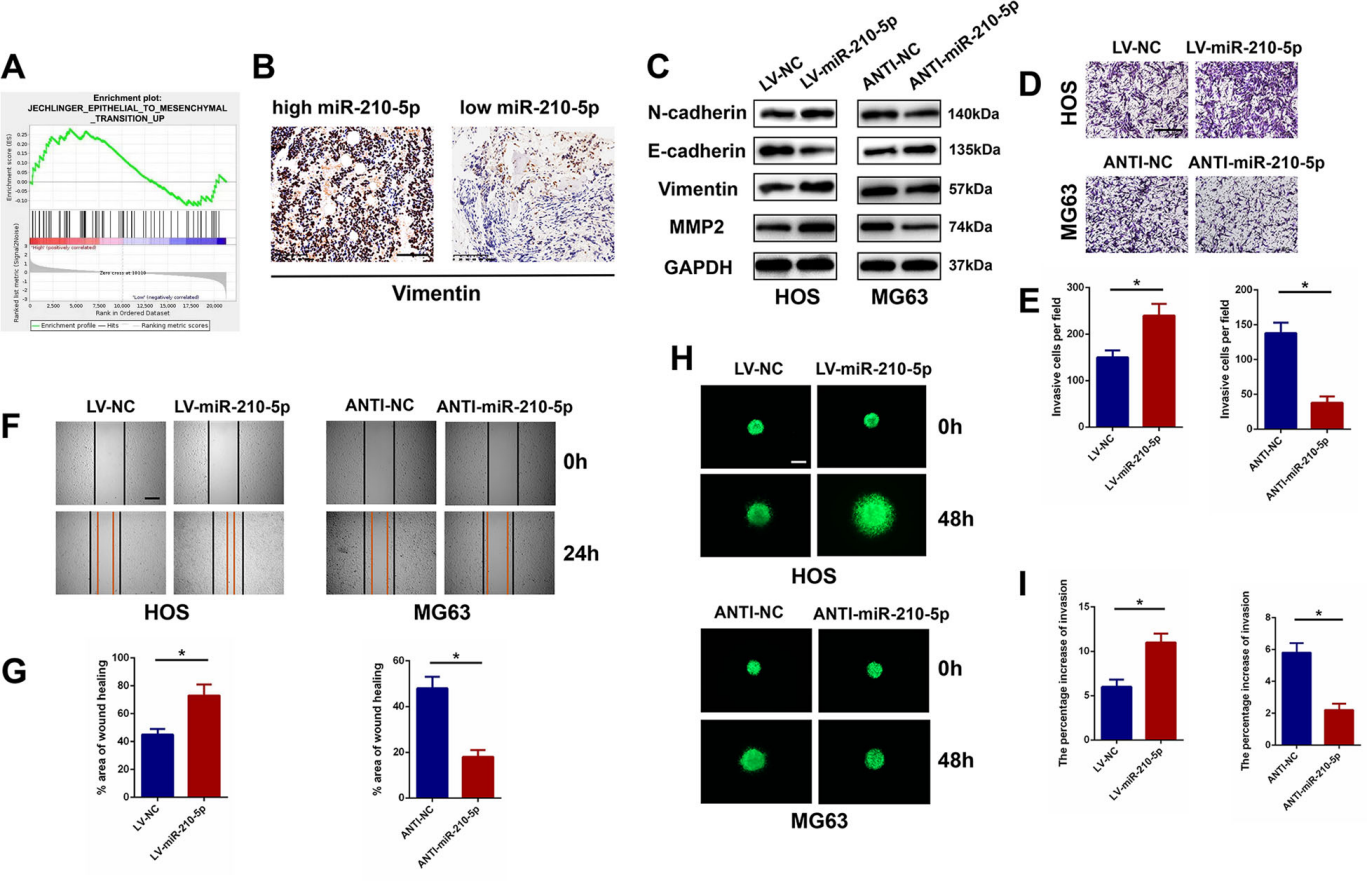
miR-210-5p promotes tumor invasion and migration in OS cells. **a** A gene set enrichment analysis (GSEA) was used to compare the high miR-210-5p group (red) with the low miR-210-5p group (blue) among the OS cohorts in the TCGA data set. Higher miR-210-5p expression was correlated with the EMT process. **b** Immunohistochemical staining of vimentin in OS tissues. Scale bar = 100 μm. **c** Western blot analysis of EMT and invasion-related protein levels in transfected HOS and MG63 cells. Protein levels on western blot were quantified by densitometry of N-cadherin, E-cadherin, vimentin, and MMP2, normalized to GAPDH. **d**, **e** Effects of miR-210-5p on invasion in vitro using the transwell invasion assay. Quantification of the transwell invasion assay is shown. Scale bar = 200 μm. **f**, **g** Wound-healing assay was performed using HOS and MG63 cells, and cell migration was measured 24 h after scratching. Scale bar = 250 μm. **h**, **i** Representative images of 3D spheroid BME cell-invasion assay in transfected HOS and MG63 cells. Quantification of 3D spheroid BME cell-invasion assay is shown. Scale bar = 250 μm.

Based on the expression level of miR-210-5p in the OS cell lines, HOS and MG63 cell lines were transfected with LV-miR-210-5p or ANTI-miR-210-5p lentivirus, respectively. The expression level after transfection was assessed using miRNA RT-PCR (Fig. 1e, f). Gene set enrichment analysis (GSEA) was performed, and it was found that miR-210-5p expression was positively correlated with EMT-associated gene signatures, which means that miR-210-5p may have an impact on the EMT process in OS (Fig. 2a). Staining of vimentin, a mesenchymal biomarker, showed that the expression level of vimentin was higher in the high miR-210-5p group (Fig. 2b). Furthermore, overexpression of miR-210-5p in HOS cells increased the expression levels of mesenchymal markers including N-cadherin, Vimentin, and MMP2, but decreased the expression of epithelial cell marker E-cadherin. In contrast, suppression of miR-210-5p in MG63 cells showed the opposite effects (Fig. 2c). A transwell invasion assay was then conducted to investigate the impact of miR-210-5p on cell invasion and migration ability in OS. As shown in Fig. 2d, overexpression of miR-210-5p significantly promoted HOS cell invasiveness, and silencing miR-210-5p attenuated MG63 cell invasiveness (Fig. 2e). A wound-healing assay was then performed, and the results demonstrated that overexpression of miR-210-5p markedly promoted the migration of HOS cells, while downregulation of miR-210-5p showed the opposite effect in MG63 cells (Fig. 2f, g). Furthermore, these results were confirmed using 3D spheroid BME cell-invasion assays (Fig. 2h, i). The effect of miR-210-5p on proliferation of the OS cell lines was evaluated by CCK-8 and colony-formation assays. It was found that upregulation or downregulation of miR-210-5p had no significant influence on cell proliferation in the first 3 days, but markedly promoted or reduced cell proliferation after 5 days in HOS and MG63 cells, which indicated that miR-210-5p could potentially promote OS cell growth (Supplementary Fig. S3). In addition, western blot and flow cytometry were performed to study the role of miR-210-5p in apoptosis in OS cells. As shown in Supplementary Fig. S4, western blot and flow cytometry results demonstrated that miR-210-5p attenuates cell apoptosis in OS cells. Taken together, these results demonstrated that miR-210-5p can enhance invasion and migration of OS cells by promoting EMT.

### miR-210-5p promotes autophagy in OS cells

**Fig. 3:**
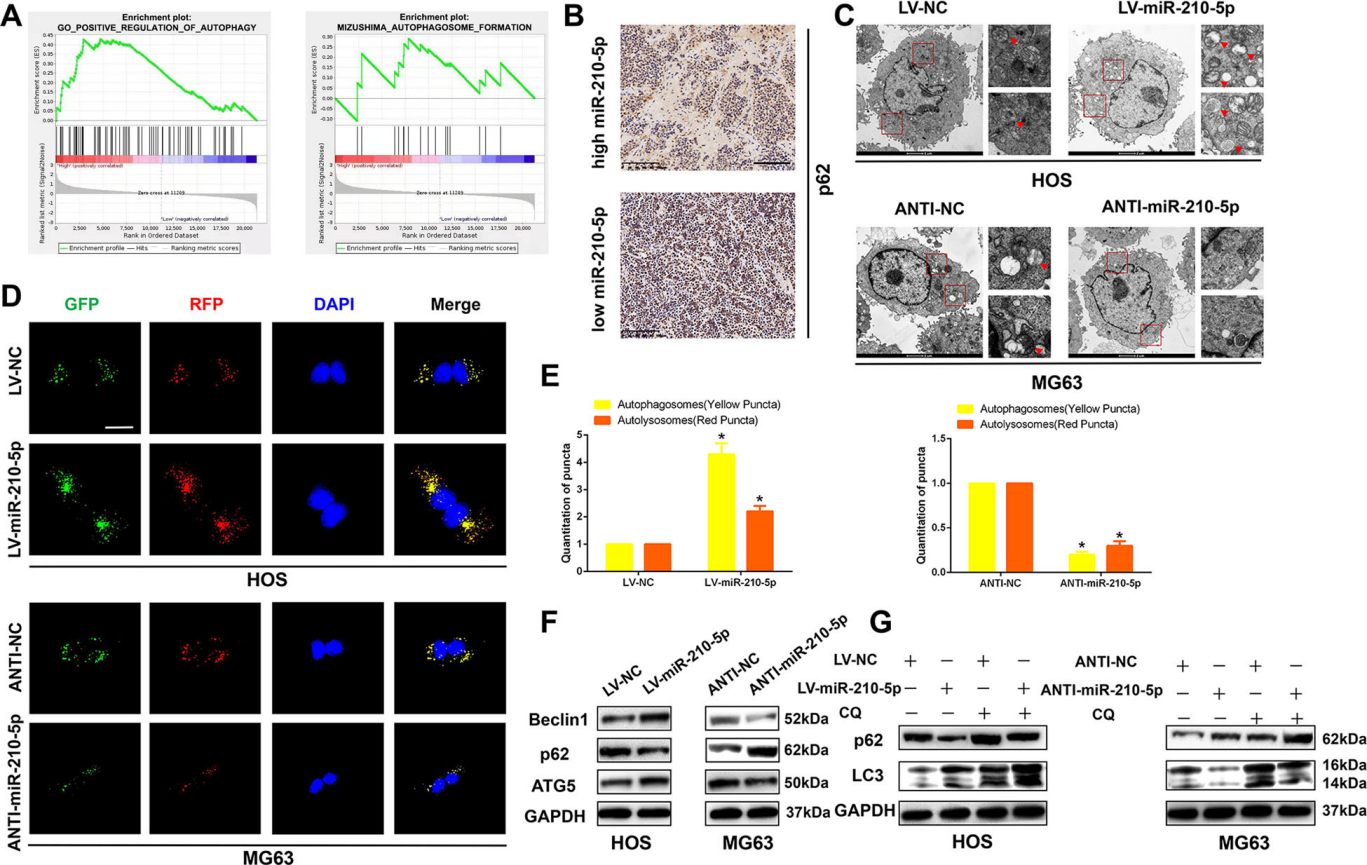
miR-210-5p promotes autophagy in OS cells. **a** Effects of miR-210-5p on the regulation of autophagy and autophagosome formation analyzed by GSEA. **b** Immunohistochemical staining of p62 in OS specimens. Scale bar = 100 μm. **c** TEM was used to detect the autophagic microstructure of transfected OS cells. Red arrows indicate a cellular autophagosome with a double-layer structure or an autolysosome generated by the fusion of an autophagosome and lysosome. **d**, **e** OS cells transfected with a GFP-mRFP-LC3 lentivirus were cultured, and yellow and red puncta were observed and counted under confocal microscopy. Scale bar = 20 μm. **f** Western blot analysis of autophagy-related protein levels including Beclin1, p62, and ATG5 in transfected OS cells. **g** Western blot analysis of LC3-II and p62 in transfected OS cells treated with the autophagy flux inhibitor chloroquine (CQ, 10 μM for 6 h).

Numerous studies have proved that autophagy has a double-edged effect on the development of tumors. Therefore, we assessed the possible association between miR-210-5p and autophagy in OS cells. A GSEA was performed to predict the relationship between miR-210-5p and autophagy. As shown in Fig. 3a, a higher miR-210-5p expression level was significantly correlated with autophagy pathway components. Thus, we performed immunohistochemistry on OS specimens and found that OS tissues with higher miR-210-5p expressed lower levels of p62 than those with lower miR-210-5p expression level (Fig. 3b). Furthermore, we detected autophagosomes using TEM and found that upregulation of miR-210-5p had favorable effects in promoting autophagy, whereas downregulation of miR-210-5p contributed to the suppression of autophagy (Fig. 3c). Moreover, to gain more insight into the relationship between miR-210-5p and autophagy, we transfected OS cell lines with GFP-mRFP-LC3 and observed the formation of autophagy flux under confocal microscopy. It was found that GFP-mRFP-LC3 dot accumulation was much more obvious when miR-210-5p was overexpressed, but decreased when miR-210-5p was knocked down (Fig. 3d, e). Moreover, western blot analysis indicated an increased level of ATG5, Beclin1, and LC3-II when miR-210-5p was overexpressed, indicating an increase in the synthesis of autophagosomes and further confirmed the above results (Fig. 3f). To further confirm these results, the lysosomal autophagy inhibitor chloroquine (CQ) was applied. As shown in Fig. 3g, when CQ was applied, the protein levels of LC3-II and p62 were significantly increased when miR-210-5p was overexpressed, which indicated that upregulation of miR-210-5p did not block autophagic flux. Collectively, these results suggested that miR-210-5p promoted autophagy in OS cells.

### PIK3R5 is downregulated and is a target gene of miR-210-5p in OS cells

**Fig. 4:**
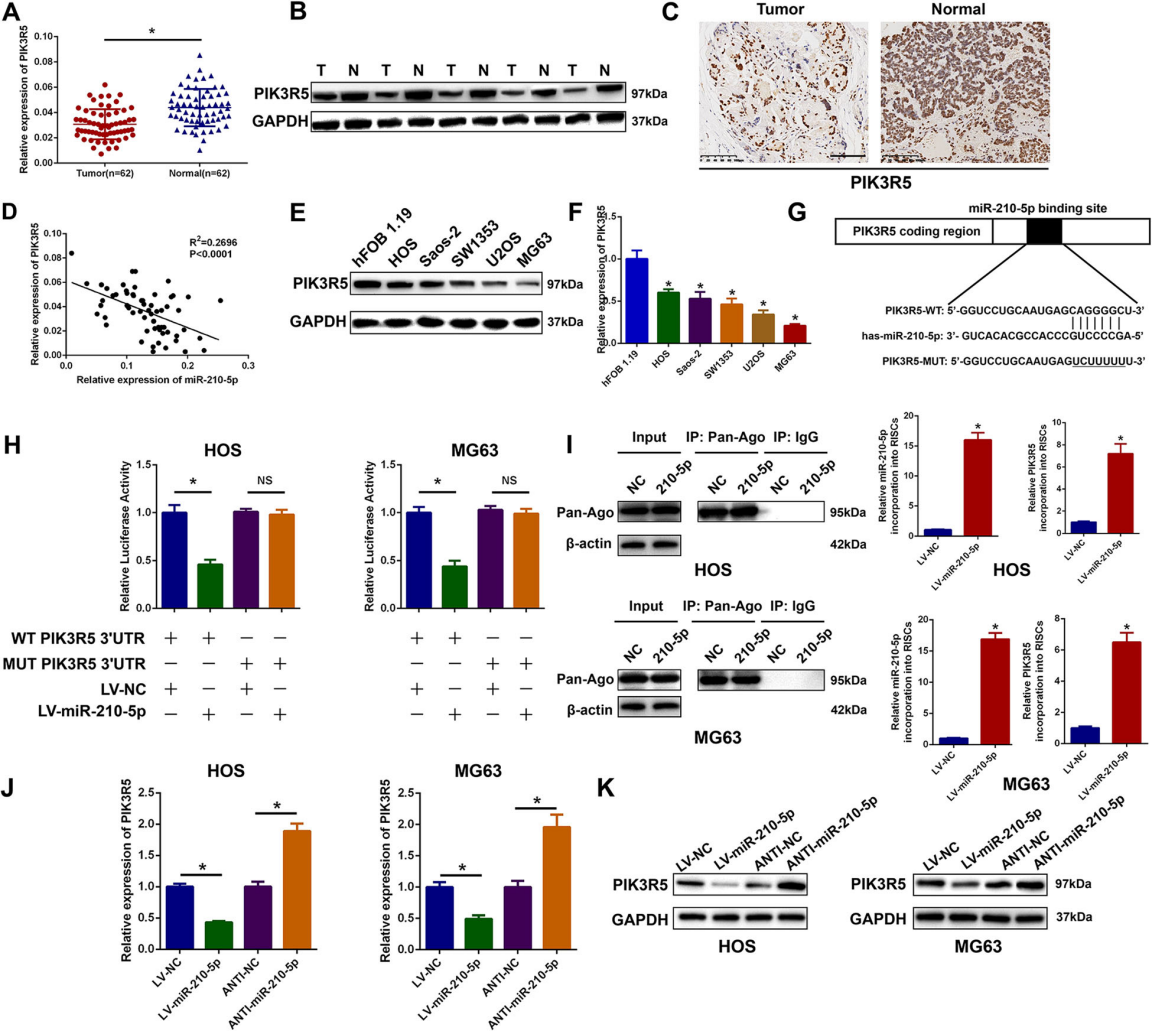
PIK3R5 is downregulated and is a target gene of miR-210-5p in OS. **a** The expression of PIK3R5 was detected in 62 pairs of OS specimens and matched adjacent normal tissues. **b** The expression of PIK3R5 was detected in five pairs of OS specimens by western blot. **c** Representative immunohistochemical staining of PIK3R5 in OS specimens and matched adjacent normal tissues. Scale bar = 100 μm. **d** Negative correlation between miR-210-5p and PIK3R5 expression in 62 pairs of OS specimens. **e**, **f** The expression of PIK3R5 in OS cells and the hFOB 1.19 cell line was detected using western blot and qRT-PCR. **g**, **h** Luciferase reporter assay was performed to confirm that miR-210-5p directly bound to the 3′-UTR region of PIK3R5. Luciferase activity was analyzed in OS cells co-transfected with LV-miR-210-5p or negative control with pGL3-PIK3R5-WT or pGL3-PIK3R5-MUT. **i** Immunoprecipitation of the Ago2/RISC (RNA-induced silencing complex) using the Pan-Ago2 antibody in HOS and MG63 cells overexpressing LV-NC or LV-miR-210-5p. IgG was used as a negative control, and β-actin was used as an internal control. qRT-PCR analysis of miR-210-5p incorporated into RISC in HOS and MG63 cells overexpressing miR-210-5p compared with the levels in the control. U6 RNA was used as an internal control. qRT-PCR of PIK3R5 incorporated into RISC in HOS and MG63 cells overexpressing miR-210-5P. GAPDH RNA was used as an internal control. **j**, **k** The expression of PIK3R5 in OS cells after alteration of miR-210-5p expression was detected by qRT-PCR and western blot.

It is recognized that miRNAs are negative regulators of mRNA translation. PIK3R5 was chosen as a potential target downstream gene of miR-210-5p by employing online databases. To confirm the relationship between miR-210-5p and PIK3R5 in OS, the expression level of PIK3R5 in 62 paired OS and adjacent normal tissues was evaluated by qRT-PCR. As shown in Fig. 4a, PIK3R5 expression in OS specimens was significantly lower than that in the matched adjacent normal tissues. The protein expression level of PIK3R5 was evaluated in five random pairs of OS tissues and adjacent normal tissues by western blot, and it was found that PIK3R5 expression was lower in OS tissues (Fig. 4b). In addition, we analyzed the expression level of PIK3R5 in online databases and confirmed that PIK3R5 expression was lower in OS specimens (Supplementary Fig. S2B). This was also confirmed by the IHC results (Fig. 4c). Furthermore, it was found that there was an inverse correlation between miR-210-5p and PIK3R5 (Fig. 4d). Moreover, PIK3R5 expression was detected in OS cell lines using western blot analysis and qRT-PCR, which revealed that the expression level of PIK3R5 was lower in OS cell lines (Fig. 4e, f). The relationship between clinicopathological features and PIK3R5 expression level was also evaluated. The expression level of PIK3R5 was significantly negatively correlated with TNM stage, lung metastasis, and tumor size (Supplementary Table S1). Furthermore, Kaplan–Meier analysis demonstrated that patients with a high level of PIK3R5 expression had a much better prognosis than those with weak expression of PIK3R5 (Supplementary Fig. S2C).

Dual-luciferase reporter assay was then performed to confirm that PIK3R5 is the target downstream of miR-210-5p. Both MUT and WT PIK3R5 3′-UTR sequences were constructed based on predictable potential binding sites using pGL3 firefly luciferase reporter plasmid (Fig. 4g). Co-transfection with LV-miR-210-5p and the pGL3-WT-PIK3R5 3′-UTR resulted in an obvious decrease in luciferase activity compared with that in the control group in both HOS and MG63 OS cell lines. However, there was no significant reduction in luciferase activity following co-transfection with pGL3-MUT-PIK3R5 3′-UTR and LV-miR-210-5p (Fig. 4h). RNA-ChIP analysis was also employed to selectively detect PIK3R5 mRNA abundance in the Ago2/RNA-induced silencing complex (RISC) after miR-210-5p overexpression. Enrichment in the levels of PIK3R5 incorporated into RISC was observed in miR-210-5p-overexpressing cells (Fig. 4i). Furthermore, we observed that miR-210-5p overexpression downregulated PIK3R5 mRNA and protein levels, and miR-210-5p silencing upregulated PIK3R5 mRNA and protein levels in HOS and MG63 cells (Fig. 4j, k).

### miR-210-5p promotes tumor invasion and migration by suppressing PIK3R5 in OS cells

**Fig. 5:**
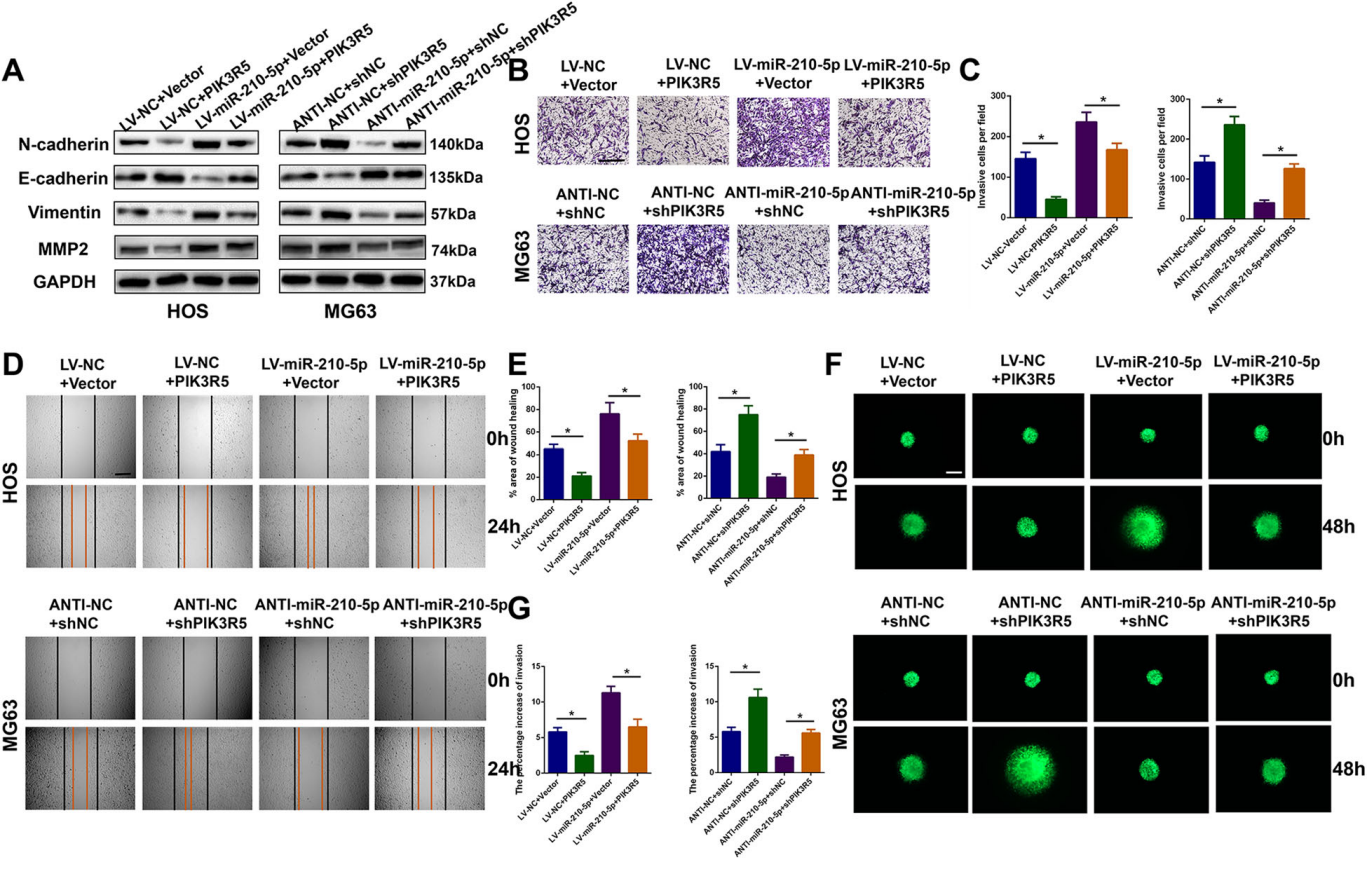
miR-210-5p promotes tumor invasion and migration by suppressing PIK3R5 in OS cells. **a**–**g** A series of gain- and loss-of-function experiments including western blot (**a**), transwell invasion assay (**b**, **c**), wound-healing assay (**d**, **e**), and 3D spheroid BME cell-invasion assay (**f**, **g**) were carried out to confirm the functional relationship between miR-210-5p and PIK3R5. **a** Western blot analysis was conducted to evaluate the EMT and invasion-related protein levels. Rescue experiments for miR-210-5p overexpression were conducted via the ectopic expression of PIK3R5 in HOS cells. Rescue experiments for miR-210-5p inhibition were conducted by downregulating PIK3R5 in MG63. **b**–**g** Rescue experiments were also conducted using the transwell invasion assay (scale bar = 200 μm), wound-healing assay (scale bar = 250 μm), and 3D spheroid BME cell-invasion assay (scale bar = 250 μm).

As the potential biological role of miR-210-5p on migration and invasion in OS cells has been demonstrated, in order to better understand the underlying mechanism of the miR-210-5p/PIK3R5 axis in OS, a series of gain- and loss-of-function experiments were performed to confirm the interaction between miR-210-5p and PIK3R5 in OS cells. First, PIK3R5 was overexpressed in HOS cells following transfection with a PIK3R5 lentivirus as well as silenced endogenous PIK3R5 in MG63 cells. Using western blot analysis, Transwell assay, wound-healing assay, and 3D spheroid BME cell-invasion assay, the results showed that co-transfection of PIK3R5 in miR-210-5p upregulated OS cells reversed the functional role of miR-210-5p in promoting tumor invasion and migration. Silencing PIK3R5 in miR-210-5p downregulated OS cells abolished the effect of miR-210-5p inhibition on OS cell invasion and migration (Fig. 5a–f). These findings indicated that miR-210-5p promoted tumor invasion and migration by suppressing PIK3R5 in OS cells.

### PIK3R5 reversed the effects of miR-210-5p on autophagy in OS cells

**Fig. 6:**
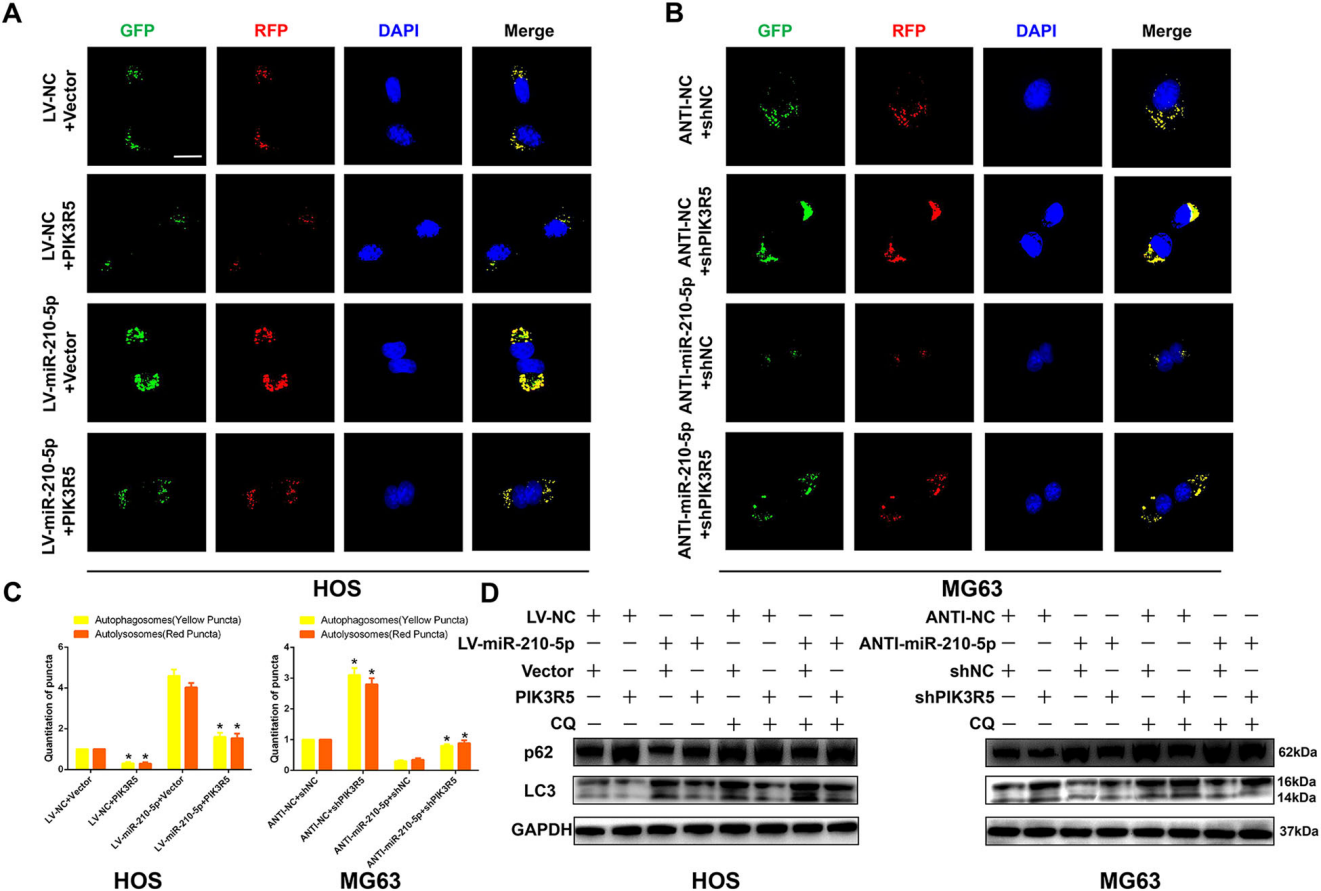
PIK3R5 reversed the effects of miR-210-5p on autophagy in OS cells. **a**–**c** HOS and MG63 cells transfected with GFP-mRFP-LC3 lentivirus were observed, and the cellular puncta were counted under confocal microscopy. Rescue experiments for miR-210-5p overexpression were conducted via the ectopic expression of PIK3R5 in HOS cells. Rescue experiments for miR-210-5p inhibition were conducted by downregulating PIK3R5 in MG63 cells. Scale bar = 20 μm. **d** Western blot analysis was used to confirm the results obtained from confocal images.

It was then determined if the effects of miR-210-5p in promoting autophagy were mediated by PIK3R5 in OS cells. As shown in Fig. 6a–d, GFP-mRFP-LC3 dot accumulation analysis and western blot analysis indicated that overexpression of PIK3R5 partially abolished the effects of miR-210-5p in promoting autophagy. Downregulation of PIK3R5 effectively restored the effects of miR-210-5p in promoting autophagy. Taken together, these results indicated that miR-210-5p promoted autophagy by suppressing the expression of PIK3R5.

### miR-210-5p-mediated autophagy contributes to miR-210-5p-induced promotion of tumor invasion and migration

**Fig. 7:**
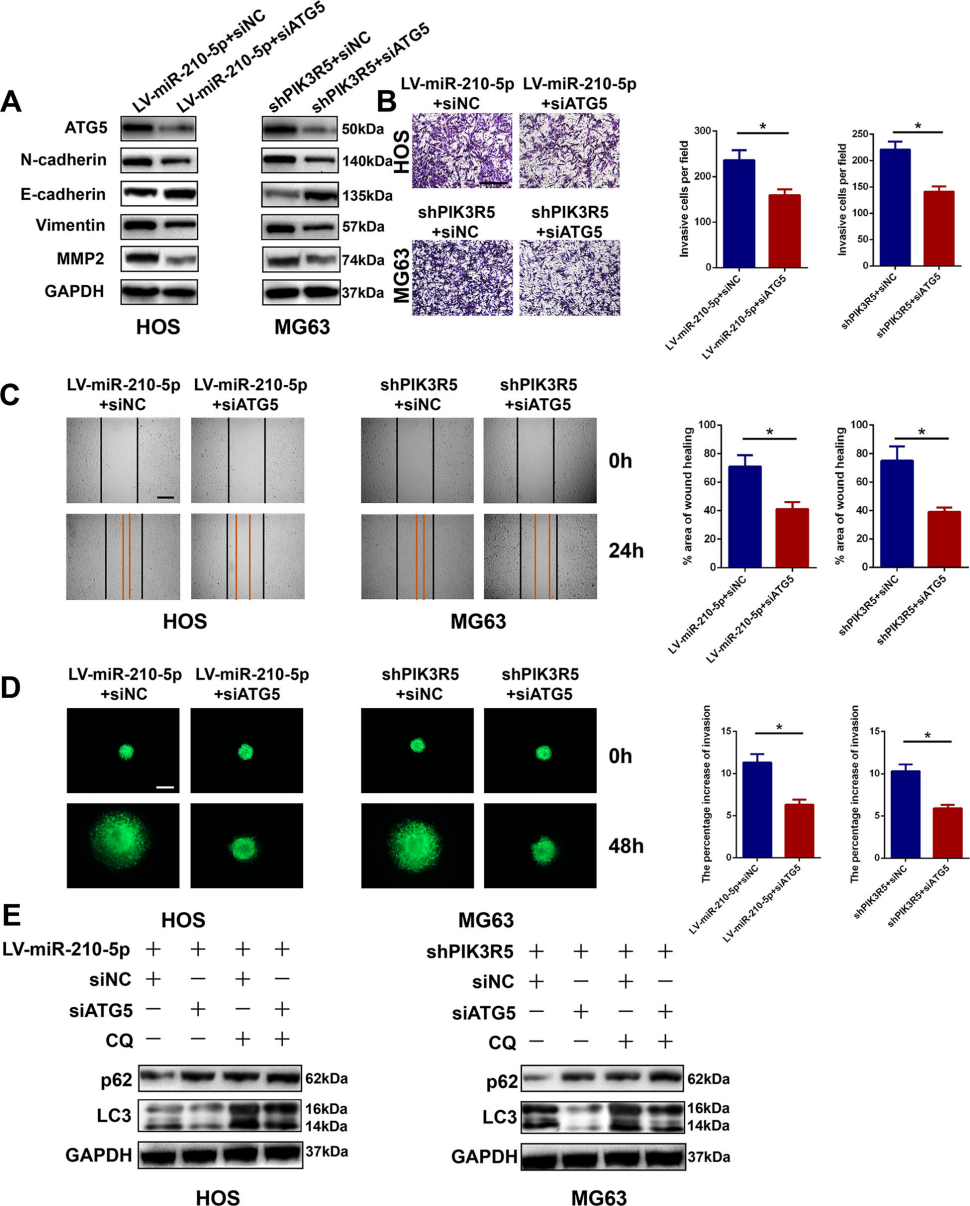
miR-210-5p-mediated autophagy contributes to miR-210-5p-induced promotion of tumor invasion and migration. **a** Silencing ATG5 significantly decreased EMT and invasion-related protein levels shown by western blot. **b**–**e** Silencing of ATG5 significantly inhibited the invasion and migration capacity of OS cells following overexpression of miR-210-5p or knockdown of PIK3R5 shown by the transwell invasion assay (**b**, scale bar = 200 μm), wound-healing assay (**c**, scale bar = 250 μm), and 3D spheroid BME cell-invasion assay (**d**, scale bar = 250 μm). **e** Western blot analysis confirmed that silencing ATG5 markedly inhibited autophagy.

To investigate whether autophagy, regulated by the miR-210-5p/PIK3R5 axis, leads to the progression of OS, we abrogated autophagy by downregulating the expression of ATG5, a crucial factor related to autophagosome elongation. Western blot analysis was performed to confirm the results (Fig. 7a). As shown in Fig. 7a–e, silencing ATG5 abolished the effects on OS invasion and migration caused by miR-210-5p overexpression or PIK3R5 knockdown. These data showed that miR-210-5p-mediated autophagy contributed to miR-210-5p-induced promotion of tumor invasion and migration.

### miR-210-5p overexpression induces autophagy by inactivating the AKT/mTOR pathway

As PIK3R5 is the regulatory subunit of phosphoinositide 3-kinase γ (PI3Kγ) that is responsible for phosphorylating membrane lipids to activate the AKT/mTOR pathway and AKT/mTOR signaling exerts important effects in regulating autophagy, we first evaluated AKT, mTOR, and their phosphorylation levels in miR-210-5p overexpressed HOS cells and miR-210-5p knocked down MG63 cells. The results showed that overexpression or knockdown of miR-210-5p did not affect the total protein levels of AKT and mTOR, but significantly affected the protein levels of their phosphorylated forms (Supplementary Fig. S5A), which indicated that the AKT/mTOR signaling pathway may be involved in miR-210-5p-induced autophagy. Meanwhile, we detected these protein levels in PIK3R5 overexpressed and knocked down OS cells. The results supported the direct role of PIK3R5 in the regulation of AKT/mTOR pathway (Supplementary Fig. S5B). In addition, SC79, an AKT activator, and GSK690693, an AKT inhibitor, were used to further identify whether the miR-210-5p-inactivated AKT/mTOR pathway regulates autophagy. As shown in Supplementary Fig. S5C, miR-210-5p overexpression inhibited the AKT/mTOR pathway, but increased the levels of LC3-II and decreased p62 expression. When SC79 was added, the AKT/mTOR signaling pathway was obviously activated, and significantly reversed the effects of miR-210-5p overexpression on autophagy in HOS cells. Following the addition of GSK690693, the AKT/mTOR signaling pathway was markedly inhibited, significantly enhancing the effects of overexpressed miR-210-5p on autophagy in HOS cells. These results were also confirmed when miR-210-5p was knocked down in MG63 cells. These results demonstrated that miR-210-5p overexpression induced autophagy by inactivating the AKT/mTOR pathway.

### miR-210-5p accelerates xenograft tumor growth and pulmonary metastasis in vivo

**Fig. 8:**
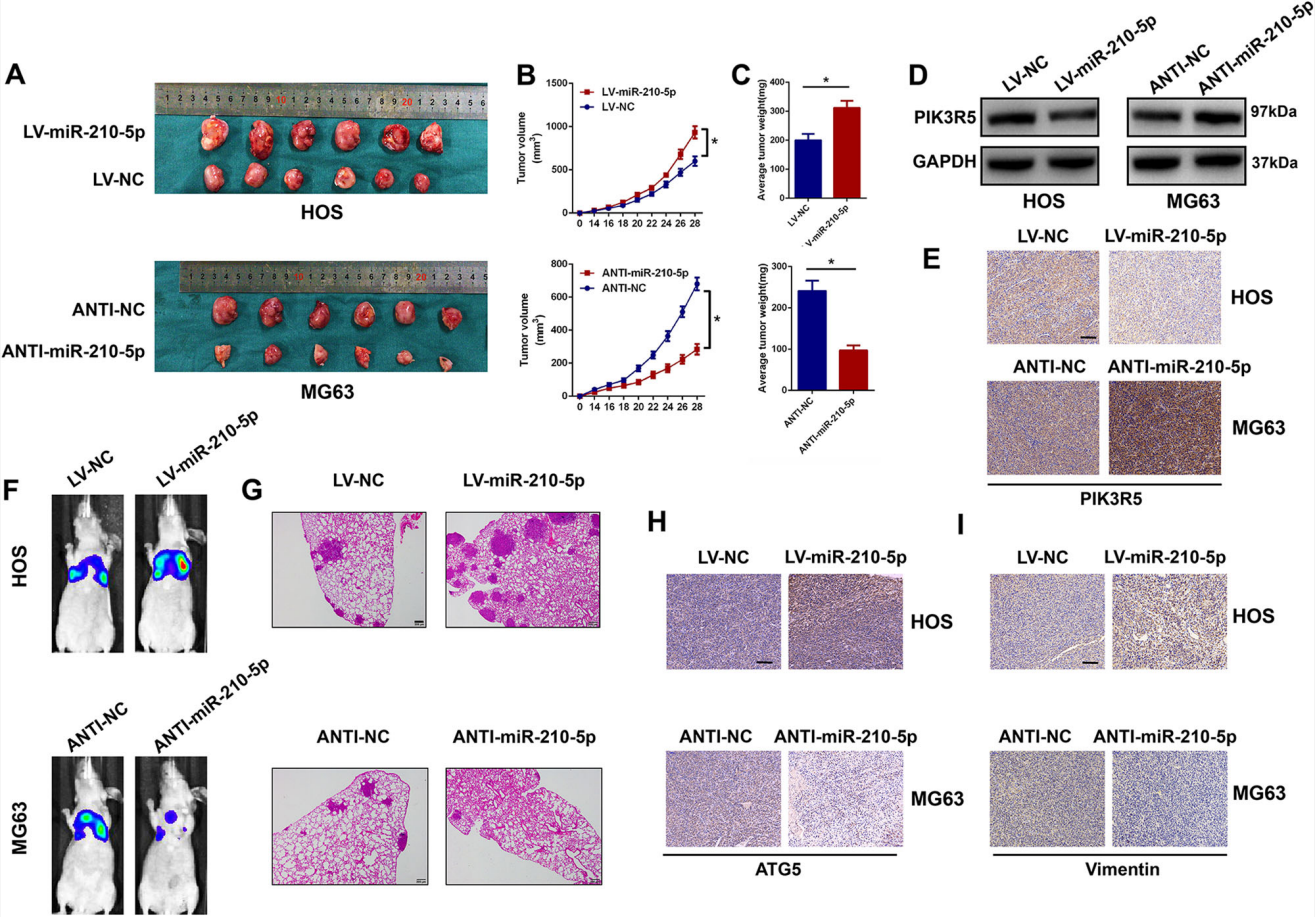
miR-210-5p accelerates xenograft tumor growth and pulmonary metastasis in vivo. **a** Images of tumors obtained from mice treated with LV-miR-210-5p and ANTI-miR-210-5p and their negative controls; *n* = 6 mice/group. **b**, **c** Tumor volume and weight were calculated. **d**, **e** Western blot and immunohistochemical analysis were performed to evaluate PIK3R5 expression in vivo. Scale bar = 100 μm. **f** Representative images of pulmonary metastases were obtained by the IVIS imaging system. **g** Representative H&E-stained lung sections from mice in different groups. Scale bar = 200 μm. **h**, **i** Immunohistochemical staining of ATG5 and vimentin in xenograft tumors. Scale bar = 100 μm.

To determine the effects of miR-210-5p on tumor progression in vivo, transfected HOS and MG63 cells were subcutaneously injected into nude mice. As shown in Fig. 8a–c, the volume and weight of tumors in the miR-210-5p overexpression group increased while those in the miR-210-5p inhibitor group decreased compared with those in the negative control groups. Furthermore, western blot and IHC analysis showed that the expression level of PIK3R5 was significantly downregulated in the implanted tumors in the LV-miR-210-5p-transfected group. In contrast, PIK3R5 expression level was markedly increased in the ANTI-miR-210-5p-transfected group (Fig. 8d, e). To further confirm the function of miR-210-5p on tumor metastasis in vivo, stably transfected cells were injected into nude mice via the tail vein. After 6 weeks, lung metastasis was significantly promoted in the LV-miR-210-5p group compared with that in the LV-NC group. In addition, lung metastasis was reduced in the ANTI-miR-210-5p group compared with the ANTI-NC group (Fig. 8f). The mice were then euthanized and lung tissues were examined by H&E staining, which showed consistent results (Fig. 8g). Furthermore, using IHC staining, the expression levels of ATG5 and vimentin were shown to be upregulated in the LV-miR-210-5p-transfected group and downregulated in the ANTI-miR-210-5p-transfected group (Fig. 8h, i). Taken together, these results demonstrated that miR-210-5p accelerated xenograft tumor growth and pulmonary metastasis in vivo.

## Discussion

OS is the most common malignant bone tumor and frequently occurs in adolescents^1,38,39^. Early formation of pulmonary metastasis is common in OS, and predicts an extremely poor prognosis^2,3^. Thus, increased attention should be paid to understanding the underlying mechanism in the progression of OS as well as identifying novel therapies to treat OS with metastasis^4,40^.

A series of complex cascades, including tumor invasion and metastasis, are affected by the tumor microenvironment and play an important role in the occurrence and development of malignant tumors^41,42^. Aberrant EMT activation is regarded as crucial in promoting tumor metastasis as it increases the viability, aggressiveness, and invasiveness of cancer cells^43^. EMT is characterized by the loss of epithelial cell connexin and increased expression of mesenchymal markers, and is thought to have an important role in tumor metastasis and progression^5–7,44^. Thus, recent studies have focused on the inhibition of EMT in malignant tumors and some studies have shown satisfactory results^45–47^.

Recently, studies have indicated that miRNAs have a marked effect on modulating cellular functions and biological processes during the progression of tumors^8,9^. Moreover, increasing evidence has demonstrated that abnormal expression of miRNAs is closely related to cell proliferation, migration, invasion, and prognosis in OS^13,14,48,49^. Although some studies have implicated miR-210-5p in various tumors, the underlying functions and mechanisms of miR-210-5p in OS have not yet been investigated. In this study, we first demonstrated that miR-210-5p was upregulated in clinical OS specimens and cell lines, which indicated that this miR may act as a tumor promoter. A series of in vitro experiments were conducted which confirmed that miR-210-5p promoted aggressive and invasion of OS cells.

Accumulating evidence has indicated that autophagy might have a double-edged role in the progression of malignant tumors by promoting cancer cell survival or leading to cancer cell death^23,24^. In order to examine the relationship between miR-210-5p and autophagy, autophagy-related experiments were performed, and the results showed that upregulation of miR-210-5p promoted autophagy in OS cells.

To further investigate the underlying mechanism of miR-210-5p in OS, bioinformatics tools were used and PIK3R5 was predicted to be a direct downstream gene of miR-210-5p. Furthermore, we confirmed this target gene using the luciferase reporter assay, RNA-ChIP analysis, and western blot analysis. It is well known that PIK3R5 is the regulatory subunit of PI3Kγ, which is responsible for phosphorylating membrane lipids to activate the AKT pathway. PI3Ks are classic key enzymes in various signal transduction pathways which modulate cell proliferation, apoptosis resistance, and tumorigenesis^50^. Moreover, PI3Ks have been proven to be vital upstream autophagy regulators, and the PI3K/AKT signaling pathway is a well-studied pathway that contributes to the activation of mTOR, which is a classic autophagy inhibitor^51,52^. Furthermore, the PI3K/AKT/mTOR signaling pathway has been reported to be involved in regulating autophagy in cancer cells^53–55^. In this study, the data showed that upregulated expression of miR-210-5p suppressed the AKT/mTOR signaling pathway and promoted autophagy in OS cells. In addition, an AKT activator and inhibitor were added, respectively, to demonstrate the relationship between the AKT/mTOR pathway and autophagy. It was shown that miR-210-5p promoted EMT by targeting PIK3R5 and thus inactivated the AKT/mTOR pathway.

It is well known that autophagy is closely related to apoptosis in cancer cells, while autophagy and EMT have been regarded as two unrelated processes for a long time. However, recent studies have demonstrated that these two crucial processes are closely correlated in a complex relationship^56–60^. It has been reported that autophagy may either inhibit or enhance EMT in different situations^56^. A previous study demonstrated that autophagy promoted pulmonary metastasis of hepatocellular carcinoma (HCC) cells^61^. Another study demonstrated that stimulation of autophagy promoted aggressive pancreatic EMT^62^. In contrast, Gugnoni et al.^30^ reported that preventing autophagy could lead to EMT and cancer metastasis. Another study also confirmed that autophagy inhibited EMT and may lead to reversion of the EMT phenotype in cancer cells^63^. In order to examine the underlying relationship between OS cells and EMT, we silenced ATG5, which is recognized to have an essential role in autophagosome formation. In this study, we found that silencing ATG5 in OS cells impaired EMT induced by miR-210-5p overexpression or knockdown of PIK3R5. Thus, our results indicated that miR-210-5p induces EMT by promoting oncogenic autophagy in OS cells.

Taken together, our findings demonstrated that miR-210-5p was upregulated and plays a crucial role in OS progression. Furthermore, it was confirmed that miR-210-5p promoted EMT by targeting PIK3R5 and thus suppressing the AKT/mTOR signaling pathway. The miR-210-5p/PIK3R5 axis mediated autophagy, which exerts oncogenic effects in OS. Moreover, our results suggested that autophagy contributes to miR-210-5p-mediated promotion of tumorigenesis and metastasis in both in vitro and in vivo models of OS. In conclusion, we demonstrated that the miR-210-5p promoted EMT by targeting PIK3R5, thereby promoting oncogenic autophagy in the progression of OS. Our findings suggested that inhibiting miR-210-5p may be a potential future treatment for OS.

## Supplementary information

Supplementary figure and table legends.

Table S1

Table S2

Figure S1

Figure S2

Figure S3

Figure S4

Figure S5
