# Supplementary material for: miR-210-5p promotes epithelial–mesenchymal transition by inhibiting PIK3R5 thereby activating oncogenic autophagy in osteosarcoma cells
Source: Cell Death Dis. 2020 Feb 5;11(2):93. doi: 10.1038/s41419-020-2270-1 (PMC7002725; doi:10.1038/s41419-020-2270-1)
Supplement: Supplementary file 1 — Supplementary figure and table legends. [file 41419_2020_2270_MOESM1_ESM.docx]

**Supplementary figure and table legends**

**Table S1.** Expression of miR-210-5p and PIK3R5 according to patients’ clinical features.

**Figure S1.** Representative X-ray, MRI and CT images of OS patients with and without pulmonary metastasis.

**Figure S2.** (A) The expression level of miR-210-5p in the OS cell line and the control from the GEO database (GSE28423). (B) The expression level of PIK3R5 in OS and the control from the ArrayExpress database (E-MEXP-3628). (C) Kaplan-Meier analysis demonstrates that patients with high PIK3R5 expression levels had a much better prognosis based on an online database (<https://hgserver1.amc.nl/cgi-bin/r2/main.cgi>).

**Figure S3. miR-210-5p has the potential to promote OS cell proliferation.** (A and B) The Cell Counting Kit-8 (CCK-8) assay and colony formation analysis revealed that the upregulation of miR-210-5p significantly promoted cell proliferation after 5 days. (C and D) Downregulation of miR-210-5p significantly inhibited proliferation of MG63 and HOS cells after 5 days.

**Figure S4. miR-210-5p reduces cell apoptosis in OS cells.** (A) Western blot analysis of apoptosis-related proteins in different groups in OS cells. (B) Flow cytometric analysis of OS cells apoptosis in different groups using Annexin-FITC/PI double staining.

**Figure S5. miR-210-5p overexpression induces autophagy by inactivating the AKT/mTOR pathway.** (A) Representative images of western blot analysis of p-AKT, AKT, p-mTOR and mTOR levels in transfected HOS and MG63 cells. (B) Representative images of western blot analysis of p-AKT, AKT, p-mTOR and mTOR levels in PIK3R5 overexpressed and knocked down OS cells. (C) Representative images of western blot analysis of p-AKT, AKT, p-mTOR, mTOR, p62 and LC3-II levels in transfected HOS and MG63 cells treated with the AKT activator SC79 and the AKT inhibitor GSK690693, respectively.
