# Supplementary material for: miR-210-5p promotes epithelial–mesenchymal transition by inhibiting PIK3R5 thereby activating oncogenic autophagy in osteosarcoma cells
Source: Cell Death Dis. 2020 Feb 5;11(2):93. doi: 10.1038/s41419-020-2270-1 (PMC7002725; doi:10.1038/s41419-020-2270-1)
Supplement: Supplementary file 2 — Table S1 [file 41419_2020_2270_MOESM2_ESM.docx]

**Table1** Expression of miR-210-5p and PIK3R5 according to patients’ clinical features

|  |  | miR-210-5p expression | |  | PIK3R5 expression | |  |
| --- | --- | --- | --- | --- | --- | --- | --- |
| Characteristics | Number | High group | Low group | P value | High group | Low group | P value |
| Age(y) |  |  |  |  |  |  |  |
| <18 | 35 | 16 | 19 | 0.63 | 17 | 18 | 0.75 |
| ≥18 | 27 | 14 | 13 |  | 12 | 15 |  |
| Gender |  |  |  |  |  |  |  |
| Female | 26 | 13 | 13 | 0.83 | 12 | 14 | 0.46 |
| Male | 36 | 17 | 19 |  | 20 | 16 |  |
| Location |  |  |  |  |  |  |  |
| Femur/Tibia | 50 | 27 | 23 | 0.80 | 24 | 26 | 0.69 |
| Elsewhere | 12 | 6 | 6 |  | 5 | 7 |  |
| TNM stage |  |  |  |  |  |  |  |
| I | 28 | 12 | 16 | 0.014^a^ | 20 | 8 | 0.002^a^ |
| II/III | 34 | 25 | 9 |  | 11 | 23 |  |
| Tumor size(cm) |  |  |  |  |  |  |  |
| <5 | 32 | 13 | 19 | 0.02^a^ | 22 | 10 | 0.005^a^ |
| ≥5 | 30 | 21 | 9 |  | 10 | 20 |  |
| Metastasis |  |  |  |  |  |  |  |
| Yes | 24 | 15 | 9 | 0.03^a^ | 7 | 17 | 0.001^a^ |
| No | 38 | 13 | 25 |  | 27 | 11 |  |
| ^a^P<0.05(Chi-square test) |  |  |  |  |  |  |  |
