# Supplementary material for: miR-210-5p promotes epithelial–mesenchymal transition by inhibiting PIK3R5 thereby activating oncogenic autophagy in osteosarcoma cells
Source: Cell Death Dis. 2020 Feb 5;11(2):93. doi: 10.1038/s41419-020-2270-1 (PMC7002725; doi:10.1038/s41419-020-2270-1)
Supplement: Supplementary file 3 — Table S2 [file 41419_2020_2270_MOESM3_ESM.pdf]

Primer sequences:

	Forward
hsa-miR-210-5p	5'-CTGTGCGTGTGACAGC-3'
U6	5'-CTCGCTTCGGCAGCACA-3'
PIK3R5	5'-GGAGGAGAGCACCAATGACATC-3'
GAPDH	5'-TAATCTTCGCCTTAATACTT-3'

Reverse
5'-GTGCAGGGTCCGAGGT-3'
5'-AACGCTTCACGAATTTGCGT-3'
5'-CAAAGCGGCAGTAGTAGAGTAGC-3'
5'-AGCCTTCATACATCTCAA-3'
