# Supplementary figures and images for: miR-210-5p promotes epithelial–mesenchymal transition by inhibiting PIK3R5 thereby activating oncogenic autophagy in osteosarcoma cells
Source: Cell Death Dis. 2020 Feb 5;11(2):93. doi: 10.1038/s41419-020-2270-1 (PMC7002725; doi:10.1038/s41419-020-2270-1)

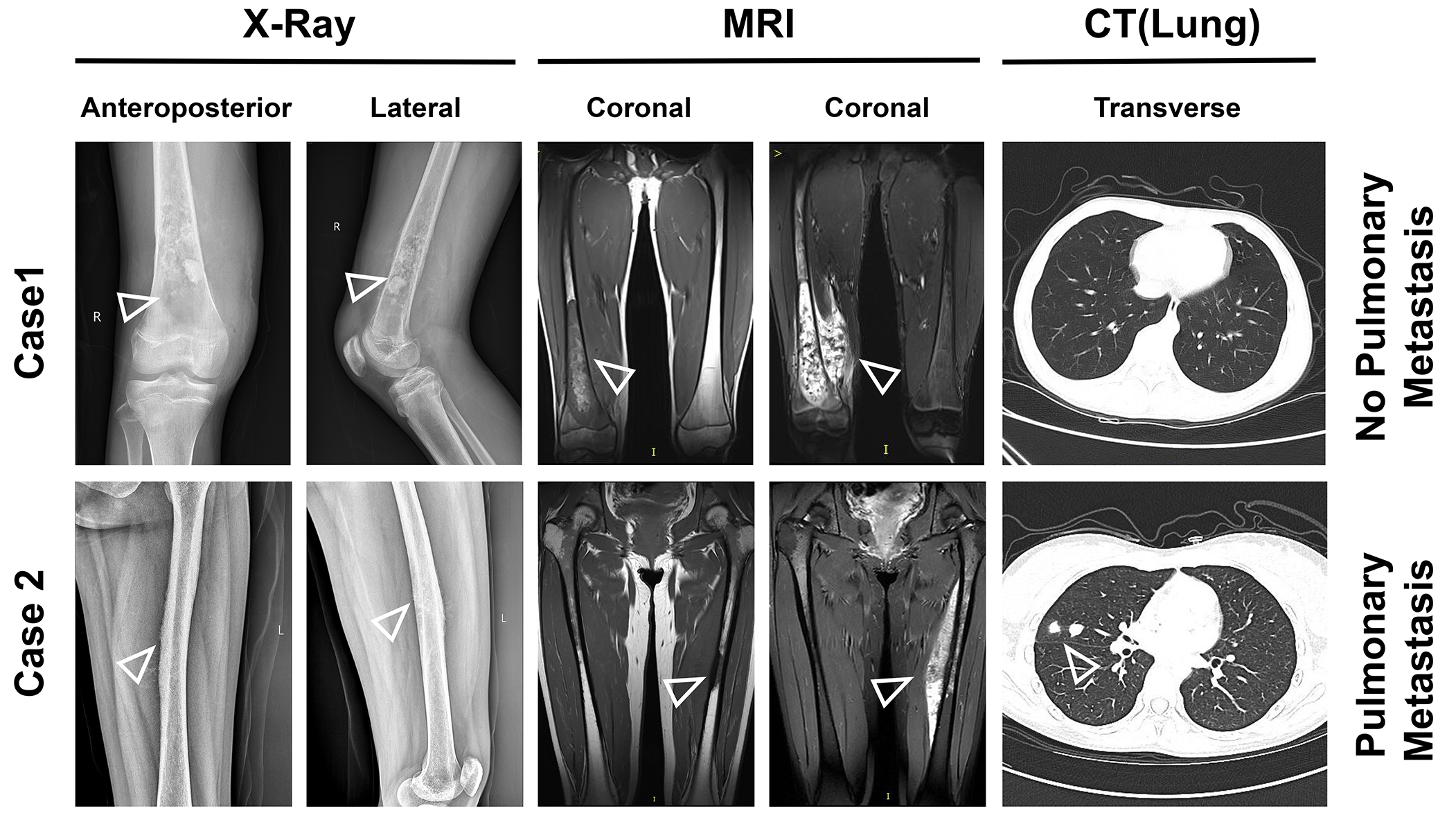

Supplement: Supplementary file 4 — Figure S1 [file 41419_2020_2270_MOESM4_ESM.tif]

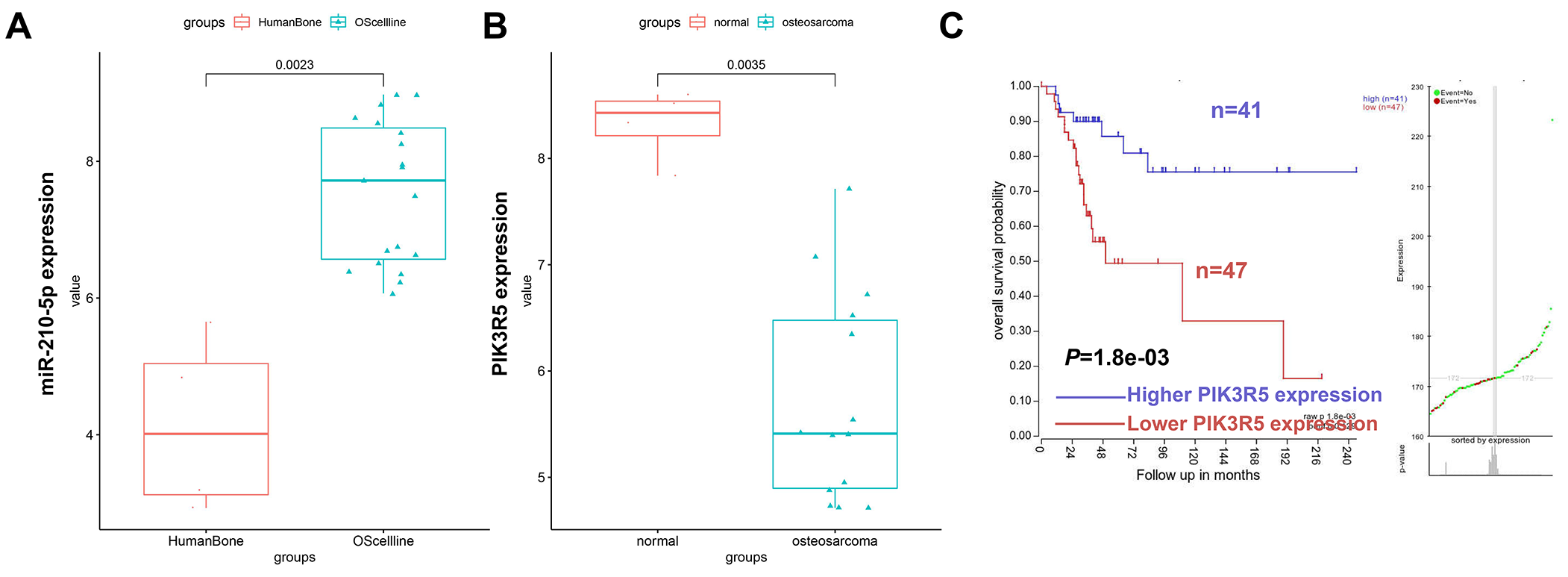

Supplement: Supplementary file 5 — Figure S2 [file 41419_2020_2270_MOESM5_ESM.tif]

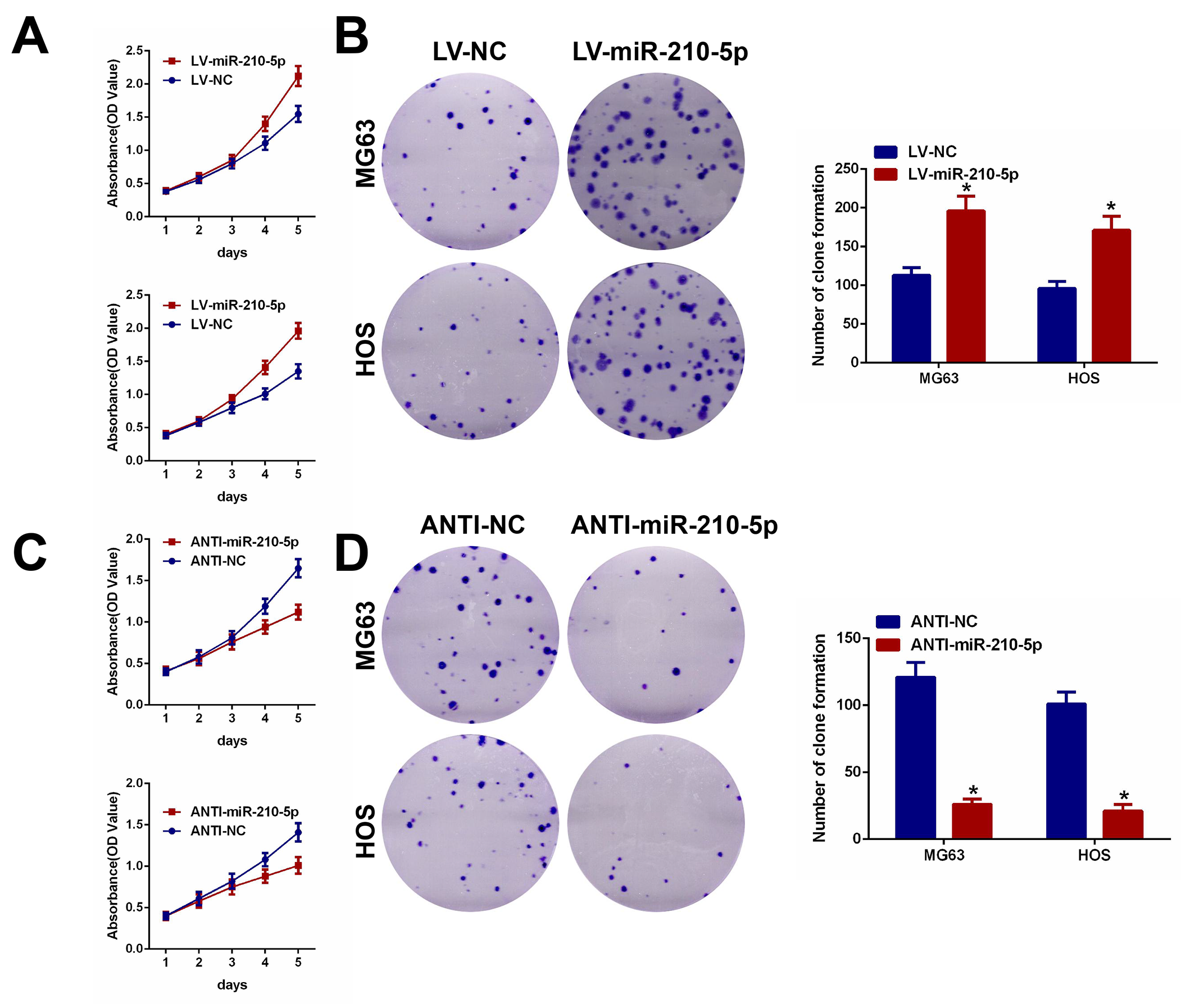

Supplement: Supplementary file 6 — Figure S3 [file 41419_2020_2270_MOESM6_ESM.tif]

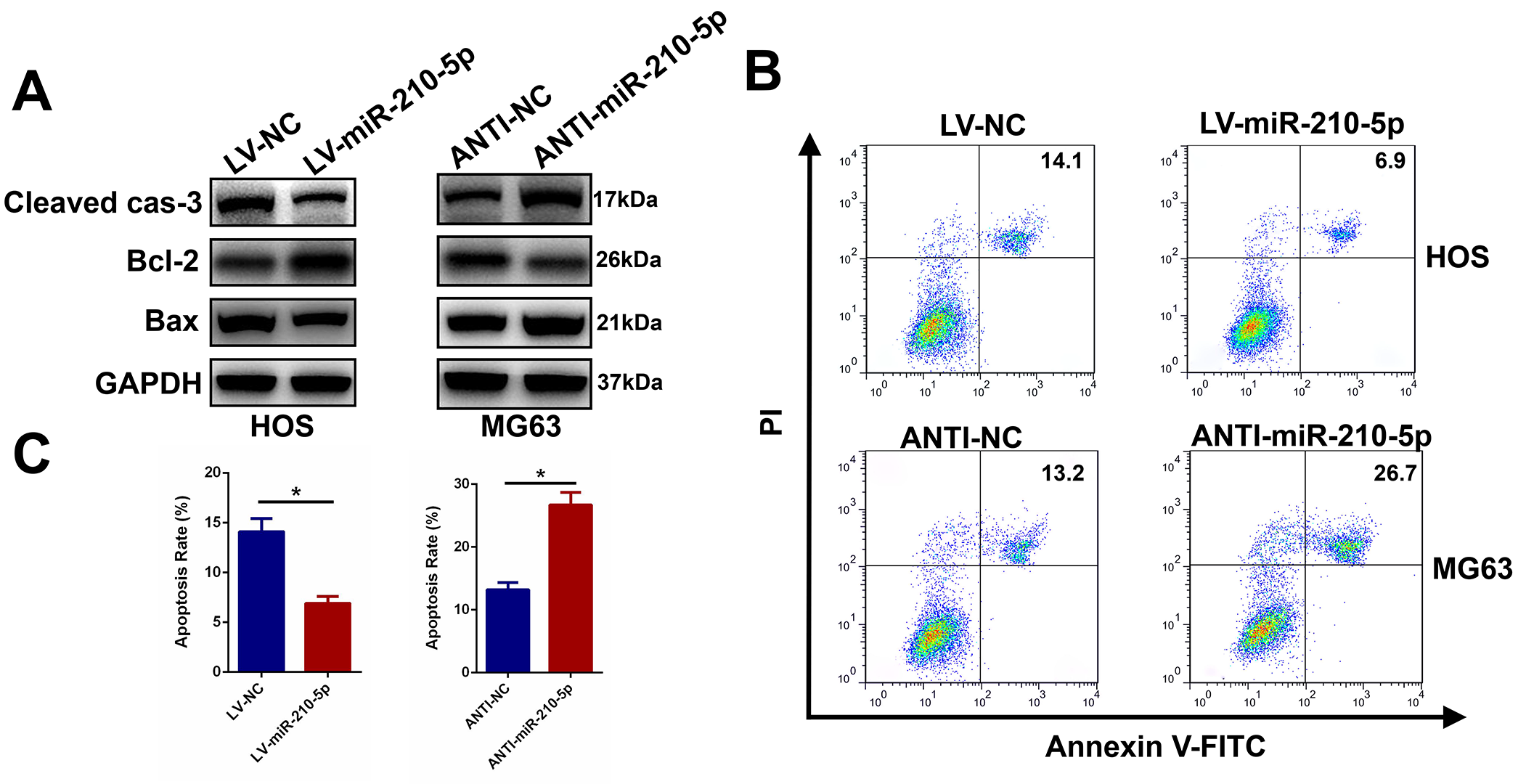

Supplement: Supplementary file 7 — Figure S4 [file 41419_2020_2270_MOESM7_ESM.tif]

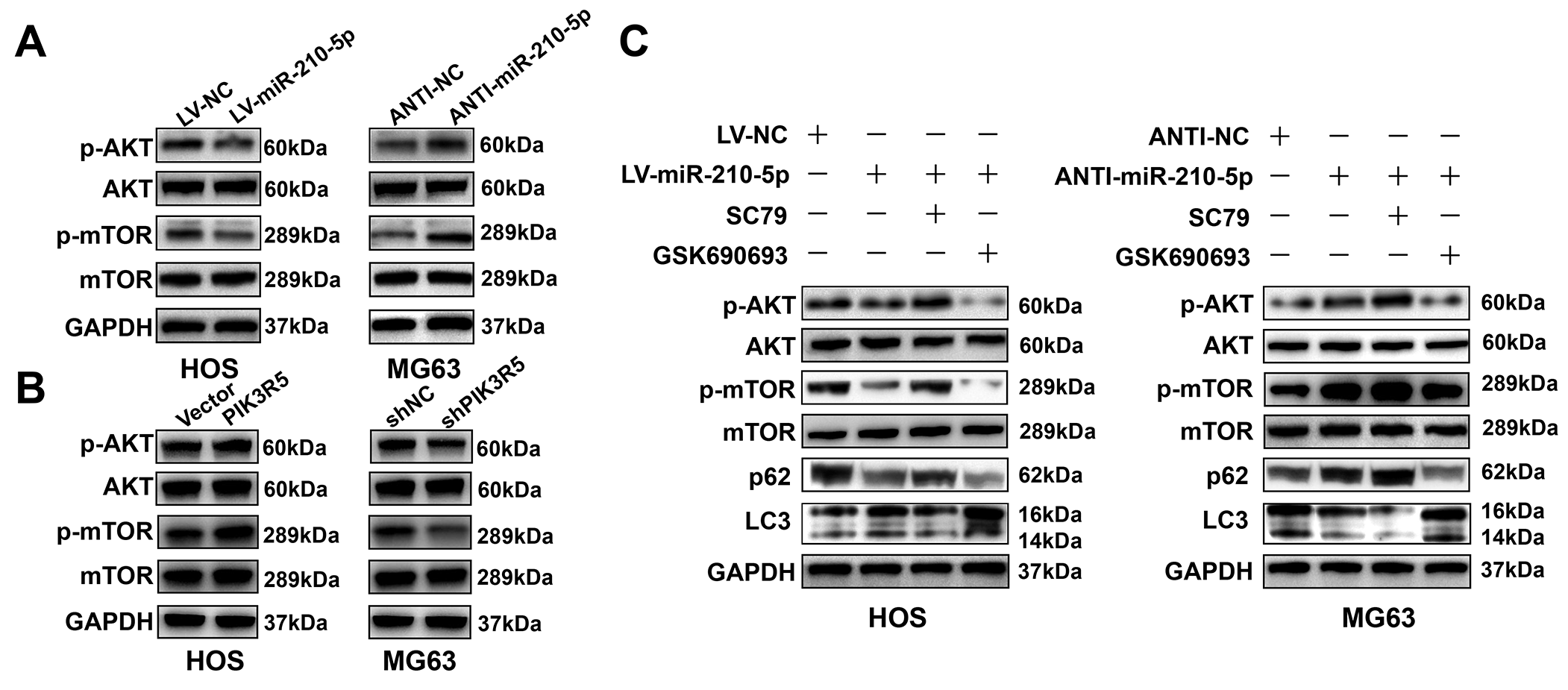

Supplement: Supplementary file 8 — Figure S5 [file 41419_2020_2270_MOESM8_ESM.tif]
